# Stochastic model of ERK-mediated progesterone receptor translocation, clustering and transcriptional activity

**DOI:** 10.1038/s41598-022-13821-x

**Published:** 2022-07-11

**Authors:** Tatiana T. Marquez-Lago, Stanly Steinberg

**Affiliations:** 1 Department of Genetics, School of Medicine, University of Alabama at Birmingham, Birmingham, AL USA; 2 Department of Mathematics and Statistics, University of New Mexico, Albuquerque, NM USA

**Keywords:** Cancer models, Biochemical reaction networks, Cellular signalling networks, Computational models

## Abstract

Progesterone receptor (PR) transcriptional activity is a key factor in the differentiation of the uterine endometrium. By consequence, progestin has been identified as an important treatment modality for endometrial cancer. PR transcriptional activity is controlled by extracellular-signal-regulated kinase (ERK) mediated phosphorylation, downstream of growth factor receptors such as EGFR. However, phosphorylation of PR also targets it for ubiquitination and destruction in the proteasome. Quantitative studies of these opposing roles are much needed toward validation of potential new progestin-based therapeutics. In this work, we propose a spatial stochastic model to study the effects of the opposing roles for PR phosphorylation on the levels of active transcription factor. Our numerical simulations confirm earlier in vitro experiments in endometrial cancer cell lines, identifying clustering as a mechanism that amplifies the ability of progesterone receptors to influence gene transcription. We additionally show the usefulness of a statistical method we developed to quantify and control variations in stochastic simulations in general biochemical systems, assisting modelers in defining minimal but meaningful numbers of simulations while guaranteeing outputs remain within a pre-defined confidence level.

## Introduction

Endometrial cancer is the most common malignancy of the female genital tract, making up 97% of all uterine cancers, and is the fourth most common cancer in women^1,2^. Progesterone, acting through its receptors PRA and PRB, is the principal steroid hormone that inhibits the growth of endometrial cancer cells^3^, through its ability to form homo- and hetero-dimers that bind to the promoters of specific genes and regulate their transcription. However, progestin therapy ultimately fails in most patients, an event that is typically linked to the loss of PR^2^. Hence one goal for endometrial cancer therapy is to maintain PR levels and activity to maintain responsiveness to progestin.

Among various factors, three seem to play a major role in controlling PR function: the nuclear localization of PRA and PRB, the presence of progestin ligand, and cross-talk with pathways stimulated by epidermal growth factor receptors (EGFR/ErbB1). In the absence of growth factor stimulation, progesterone-induced nuclear localization of PRB is a relatively inefficient and slow process that takes place over approximately 30 min^4^. In contrast, EGFR activation induces progesterone-independent translocation of PRB to the nucleus much more rapidly, typically within 5 min^4^. This mechanism of PRB nuclear translocation can be blocked by protein kinase inhibitors, as explained by events downstream of growth factor receptor signaling: the EGFR cascade results in the activation of ERK protein kinases, which in turn phosphorylate PR on serine 294 in response to progestin ligand. Phosphorylation permits PRB targeting to the nucleus and enhances its affinity for ligand, improving the likelihood that PRB will form active dimers and upregulate target gene transcription^5^. However, the same phosphorylation event results in PR ubiquitination and destruction in the proteasome^6^. Thus, phosphorylation of PR by activated ERK both induces the optimal transcriptional conformation of PR and targets the molecule for proteolysis^4^.

Phosphorylation of PR by ERK in response to constitutively high levels of EGF/ErbB1 signaling, a condition present in some tumors, may limit the long-term effectiveness of progestin therapy in the treatment of endometrial cancer by down-regulating receptor levels. Consequently, the primary objective of this study is to model ERK-mediated regulation of PR levels and activity. We also consider other known characteristics of these nuclear receptors, such as clustering within the nucleus^1,7^ and the differential distributions of resting PRA and PRB. For this, it has been previously shown that approximately 50% of PRB is cytoplasmic in the absence of progesterone, while PRA is nearly all nuclear whether or not ligand is present^8^. With the addition of progesterone ligand, however, cytoplasmic PRB shuttles into the nucleus, a process that appears to require receptor phosphorylation^6^. Our model includes the translocation of PRB into the nucleus and, by numerically fine-tuning parameters, we identify conditions that are predicted to maintain optimal levels of activated PR, theoretically allowing for extended progestin treatment. We also find that fine-tuning EGFR activity with a tyrosine kinase inhibitor along with PR ligand-induced clustering increases the ability of progesterone receptors to regulate gene transcription.

Our modeling workbench was used to find conditions that will optimize the production of transcription factor, depending on cell cytology conditions. We utilize several tools for this, including coarse-grain numerical solutions, a fine-grain spatial stochastic simulator, and tools for testing the sensitivity of the system to changes in the parameters. The fine-grain stochastic models were created in ChemCell, a whole-cell spatially and temporally resolved stochastic simulator for cell chemistry that treats proteins, protein complexes and organic molecules as individual particles diffusing via Brownian motion, where each particle has a spatial position and is assigned a species type^9^. We purposely present our original simulations with ChemCell, in memoriam of Dr. Alex Slepoy^10^, an early collaborator in this project. However, we note we also validated our results using Smoldyn^11^, a broadly used spatial simulator. The coarse grain model is deterministic and is based on solving chemical mass balance equations, allowing estimation of cascade parameters and consequently reducing the number of costly stochastic simulations. We additionally performed kinetic analysis and developed simple models for approximating translocation processes between cellular compartments. Lastly, we introduce a simple yet novel method for measuring variations in stochastic simulations based on the Bonferroni’s inequality^12^, facilitating easy determination of the number of simulations necessary to remain within a pre-determined level of accuracy. There are alternative methods to split spatial models into parts, such as^13^, but depending on the simulator, and especially when dealing with disparate chemical and diffusion rates, it can prove useful to predetermine the number of simulations necessary to capture essential dynamics of a stochastic system within a predefined level of confidence, as presented here.

## Materials and methods

### Cell and nuclear dimensions

Endometrial cell diameters were estimated for suspended Hec50co cells using the Vi-cell XR cell viability analyzer (Beckman-Coulter). We selected Hec50co cells for our model because they faithfully replicate molecular characteristics of type II endometrial cancers^14^. For determination of cytoplasmic and nuclear volumes, Hec50co cells were fixed in 2% glutaraldehyde, embedded in epon and thin sections prepared for routine transmission electron microscopy. Images were acquired on a Hitachi H7500 equipped with a digital camera. Hec50co cells were initially obtained from Kimberley Leslie and subsequently analysed in the Oliver laboratory (cf. Acknowledgments). Dimensions of cellular compartments were estimated using ImageJ (courtesy of Tomas Mazel, cf. Acknowledgments).

### Cell-signaling models

The choice for a particular modeling approach depends on features characteristic to each biological system, such as molecular concentrations, distribution, the types of reactions (diffusion-limited or reaction-limited) and whether discreteness and internal noise have no noticeable macroscopic effects, among various factors. The most detailed cell signaling models are both stochastic and spatial and are numerically represented by either stochastic partial differential equations or studied by spatiotemporal simulations representing stochastic particles. The latter generally perform single particle tracking and contain information on the places and times where all molecular reactions occur. One example of such a simulation platform is ChemCell^9^, used here in tribute to Alex Slepoy, but we note there are other simulation algorithms that perform the same and even more elaborate spatiotemporal simulations such as Smoldyn^11,15–17^, MCell^10,18,19^, and Green’s function reaction dynamics^20–25^, while being continuously maintained.

We split our model into two parts, accordingly. The first part refers to reactions involving large homogeneous molecular concentrations, followed by a compartment translocation event in a relatively large time span, represented by a deterministic non-spatial model. These results feed the second model in the form of rate constants and validate the temporal dynamics of reactions occurring upstream of the formation of active PR clusters. This second part of our model considers spatially inhomogeneous reaction–diffusion events. Both parts are explained in detail in the “Results” section.

### Stochastic variation envelopes

Since the output of general stochastic simulations can show considerable variability, it is advisable to perform a sufficiently large number of simulations and then report on the mean of their outputs as the result of a model. However, it is at least as important to quantify the variability in the results of the simulations, and to determine a meaningful number of simulations that will be required to maintain a desired level of accuracy, throughout the simulation time span.

To that effect, we introduce a straightforward method that can be used to construct envelopes for general stochastic simulations. Such envelopes contain the outputs of simulations at all time steps, with a pre-specified confidence level. A more detailed description, justification and extensions of this method can be found in^12^. Here, we present a summary the methodology, within the context of chemical kinetics in cells. However, it should be kept in mind that the exact same methodology can be used for any application that requires stochastic simulations.

Let us first assume that any stochastic modeling program is run *M* times under identical conditions (except for the pseudo-random values used as seeds to the code) and outputs concentration results ${X}_{j}^{i}$ for each *j*-th run at time *t* = *t*_*i*_, for $1\le i\le N$ and $1\le j\le M$. These concentration results, or numbers of molecules, are obtained for each chemical species. Next, assume that the results can be modeled by random variables with finite population mean ${\mu }_{i}$ and standard deviation ${\sigma }_{i}$, both of which can be approximated by the sample mean $\overline{{X }_{i}}$ and standard deviation $\overline{{S }_{i}}$ at every time step *t* = *t*_*i*_.

The Central Limit Theorem (CLT) states that, in random sampling from an *arbitrary* population with mean $\mu$ and standard deviation $\sigma$, if the sample size *M* is large enough, we will have that the standard normal random variable Z is such that

$$Z= \frac{\overline{X }- \mu }{\sigma /\sqrt{M}} \sim N(\mathrm{0,1})$$

where *N(0,1)* denotes the normal distribution with mean $\mu =0$ and standard deviation $\sigma =1$. This means that, whether the population original distribution is continuous or discrete, symmetric or asymmetric, normal or not, as long as the population variance is finite, the distribution of the sample mean $\overline{X }$ is nearly normal if the sample size *M* is large.

Having this in mind, we can use the CLT to construct variation estimates for any set of stochastic simulations. To say something about their outputs, one must compute their average at all desired time steps which, in turn, are guaranteed to be normally distributed if the number of simulations is large enough. A confidence interval is then defined in terms of the inverse survival function for the normal distribution, where $\alpha \epsilon [\mathrm{0,1}]$ is the confidence level and can be chosen at will. So, the interval

$$\left( {\overline{X}_{i} - Z_{\alpha /2} \frac{{\overline{S}_{i} }}{M},\overline{X}_{i} + Z_{\alpha /2} \frac{{\overline{S}_{i} }}{M}} \right)$$

will contain the unknown population mean $\mu$ approximately $\left(1-\alpha \right)100\%$ of the times.

The question now is how to construct a common variation envelope for all time steps, namely considering the whole simulation. For such a purpose, we will use a statistical tool commonly known as Bonferroni’s inequality, which performs multiple sample comparisons with heteroscedasticity, i.e. under the assumption where the standard deviation cannot be considered to be uniform for all samples^26,27^.

Bonferroni's inequality states that the probability of occurrence of one event of out a possible set of events is no more than the sum of the probabilities for the individual events. Alternatively, it can be stated as

$$P\left( {\bigcap\limits_{{i = 1}}^{M} {A_{i} } } \right) \ge 1 - \sum\limits_{{i = 1}}^{M} {P\left( {\bar{A}_{i} } \right)}$$

where ${A}_{i}$ denotes any event, and ${\overline{A} }_{i}$ is its complement. Following this idea, one can construct an interval at each time point *t*_*i*_, $1 \le i\le N$, with a confidence level of $1-\alpha /N$, ensuring that the overall confidence is of at least $1-\alpha$. In other words, Bonferroni’s inequality implies that the probability *P* of the mean ${\mu }_{i}$ being in the $1-\alpha /N$ confidence interval at *N* time points satisfies

$$P\left[ {\bigcap\limits_{i = 1}^{N} {\left( {\overline{X}_{i} - Z_{{{\raise0.7ex\hbox{$\alpha $} \!\mathord{\left/ {\vphantom {\alpha {2N}}}\right.\kern-\nulldelimiterspace} \!\lower0.7ex\hbox{${2N}$}}}} \frac{{\overline{S}_{i} }}{M} , \overline{X}_{i} + Z_{{{\raise0.7ex\hbox{$\alpha $} \!\mathord{\left/ {\vphantom {\alpha {2N}}}\right.\kern-\nulldelimiterspace} \!\lower0.7ex\hbox{${2N}$}}}} \frac{{\overline{S}_{i} }}{M}} \right)} } \right] \ge 1 - \sum\limits_{i = 1}^{N} {\frac{\alpha }{N} } = 1 - \alpha$$

where these *N* confidence intervals are called a confidence envelope.

There are many ways in which the growth of the confidence envelope can be used. One is to guarantee fluctuations remain within a certain range at a certain confidence level, but another one is to calculate the number of simulation steps that would be necessary to remain within a pre-defined accuracy level. A very simple and efficient way is to construct the confidence envelopes based on a subset of the original time steps. Ideally, such time steps should be relevant (e.g. experimental measures, or points in which there is considerable variability), in order to ensure that the simulations are statistically correct and reflect what one observes from the biology, controlling for variability, else re-fine time steps in line with desired level of accuracy.

### Deterministic coarse grain simulations and sensitivity analysis

Spatially resolved stochastic simulations can be computationally very expensive and, depending on molecular concentrations and reaction–diffusion timescales, single simulations may be obtained after several CPU days—if feasible at all. Accordingly, in some instances, and solely when retaining spatial resolution is not necessary, one can opt for less costly deterministic models. In this case, numerical solution of the resulting ordinary differential equations was done with Maple and Matlab. Sensitivity analyses were also carried out and used to monitor the change of the output with respect to reaction rates and to explore possible locations of feedback loops within the reaction cascade. As a result of our sensitivity analysis, we labeled all rates deemed not to affect the general output of the simulation as “flexible” (see Table 1). This term basically means that, compared to chemical events involving equivalent reactants, a variation of up to 2 orders of magnitude increasing or decreasing a rate did not affect the output of the signaling pathway simulation as a whole. Overall, for such reactions and for numerical purposes, we noticed there is a window of variability of 4 orders of magnitude in certain kinetic parameters. This is extremely convenient, considering exact kinetic rates for some of the reactions are quite hard to measure or find in the literature. With no apparent “switches” or bistability in this system, we then used these idealized kinetic rates and performed sets of stochastic simulations, to account for scenarios of molecular clustering.

**Table 1:** Concentrations of molecular species, diffusion, and reaction rates.

Parameter	Value	References
EGFR	50,000–100,000 per cell	Measured experimentally and^29^
ERK	50,000–100,000 per cell	^30^ and^36^
Progesterone (p4)	10^−9^–10^−6^ molar, and we used a molarity of 10^–7^ for all simulations	^32^ and^37^
Progesterone receptors	10,000–12,000 per cell	^34^ and^35^
Ubiquitin	In excess (not rate limiting)	^33^
Diffusion inside cytoplasm	10^−8^ cm^2^/s	^31^ and^37^
Diffusion inside nucleus	10^–9^ cm^2^/s	^31^ and^37^
k_1_	1	Flexible^12^, and explained in text
k_i_, ‘i’ ≠ 1, 2, 4, 6, 7	10^10^	^12^ and explained in text

### Parameter estimations

Estimates for compartmental volumes, numbers of molecules/concentrations and rate constants, were based on calculations and references reported in Tables 1 and 2, unless otherwise specified. Values reported in Table 1 are based on^12,28–36^. To convert from molarity units to numbers of molecules inside a compartment, values were multiplied by the volume of the compartment in units of liters, times Avogadro's constant. Accordingly, there can be 522–522,100 molecules for progesterone ligand from the results reported in Tables 1 and 2, as in^31^, and the concentration of the progesterone ligand (p4) ranges between 1 and 10 ng/mL, equivalent to concentrations ranging between 10^–6^ and 10^–9^ M in Hec50co cells, our reference system for modelling. An average molarity of 10^–7^ M was adopted for all simulations. All other species concentrations were obtained directly from the references reported in Table 1.

**Table 2:** Dimensions and volumes for cellular compartments.

Method	Cell dimensions/diameter ($\mathrm{\mu m}$)	Cell volume (${\mathrm{\mu m}}^{3}$)	Nuclear dimensions/diameter ($\mathrm{\mu m}$)	Nuclear volume (${\mathrm{\mu m}}^{3}$)	N
Cell counter	17.7 ± 2.1	2900	–	–	1930
Electron microscopy	18.2 ± 3.4	3130	11.2	727	4
Confocal microscopy	19	3611	12.8	1100	11
Histology	22.2 × 11.8 × 11.8	3100	17.9 × 9.6 × 9.6	870	2027

**Figure 1:**
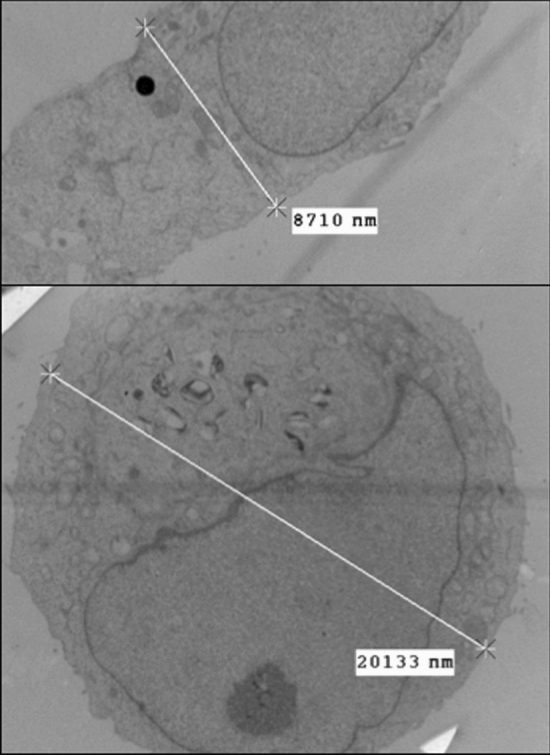
Morphometric analysis of electron micrographs taken of ultra-thin sections of Hec50co endometrial cells. Volumes for each simulation cellular compartment (cytoplasm, nucleus) were determined from estimations of suspended Hec50co cells.

Volumes for each cellular compartment (cytoplasm, nucleus) were determined by morphometric analysis of electron micrographs taken of ultra-thin sections of Hec50co endometrial cells (Fig. 1). A diffusion coefficient of 10^–8^ cm^2^/s was assumed for molecules inside the cytoplasm, while a coefficient of 10^–9^ cm^2^/s was assumed for molecules inside the nucleus, in accordance with^30,36,37^.

### Consent for publication

Both authors consent to the publication of this work.

## Results

**Figure 2:**
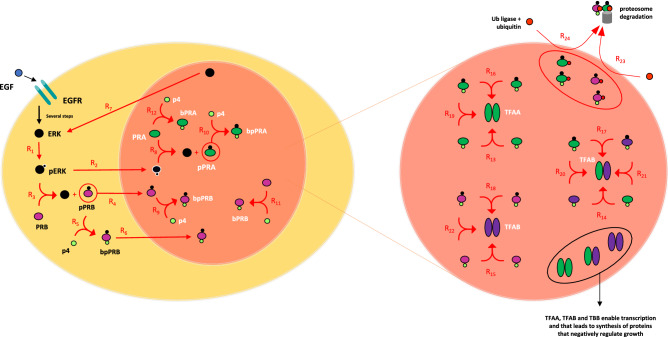
Illustration of the model summarizing key aspects of progesterone receptor regulation. Approximately 50% of PRB is cytoplasmic in the absence of progesterone, while PRA is considered to be 100% resident in the nucleus whether or not ligand is present^8^. Two ligand binding steps are required to maximally support PRB shuttling into the nucleus where it can form active dimerized transcription factor. In one step, growth factors bind to surface receptors, such as the EGFR, leading to the activation of the MAP kinase family members, ERK1 and ERK2. Activated ERK phosphorylates PRB. Cytosolic, phosphorylated PRB binds progesterone and translocate to the nucleus^6^. Ligand binding at this stage is not rate-limiting. On one hand, ligand binding is a relatively fast event (compared to the expected time for PR translocation). On the other, growth factors may bypass ligand activation by inducing rapid translocation to the nucleus, with ligand concentrations that are normally too low to stimulate activation without other stimuli^4^. PRA is also a substrate for ERK phosphorylation, once active MAP kinase translocates to the nucleus. Nuclear, ligand-bound progesterone receptors form hetero- and homodimers and, in endometrial tissue, stimulate transcription of genes that mediate differentiation and growth arrest. PRA and PRB are also substrates for ubiquitination, targeting receptors for translocation to the cytosol and proteosomal degradation.

An illustration of the model summarizing key aspects of progesterone receptor regulation is presented in Fig. 2 and described in the legend. Once in the nucleus, in our model PR is assumed to dimerize to bind to DNA and to activate gene transcription. While nuclear translocation of PR monomers occurs by either addition of ligand or ERK activation^4,8^ in both cancer and normal cells, the formation of active PR dimers and their subsequent spatial organization into clusters relies heavily on serum progesterone^7^, solely in normal cells. The latter has been confirmed by experiments showing formation of clusters following progesterone cycles. In addition, in endometrial cancers, normal tissue tends to have uniform PR distribution, whereas adjacent cancerous cells feature clusters, confirming differential reliance for PR spatial organization between normal and cancerous tissues in the same systemic environment^7^.

For purposes of our model, we term a ligand-bound PR dimer the transcription factor (TF). Note that due to the presence of two isoforms of PR that can form both homo- and heterodimers, TF can occur in three varieties, TFAA, TFAB and TFBB. Once dimers are formed, all three distinct types of TF are potential substrates for ubiquitination and subsequent proteasomal destruction^4,6^. Of note, the rate at which this appears to happen is much slower to that of ERK-mediated ubiquitination of PR. Additionally, the formation of clusters, at least in the early stages, is not linked to PR degradation^7^.

### Rapid loss of active dimers due to ubiquitination and degradation

The first simulation model explores PRB and ERK translocation in response to rapid ERK activation, compared to slow ligand-dependent translocation in scenarios of blocked EGFR activity. Due to both spatial homogeneity and high molecular concentrations, our simulations in this first part of the model are solely deterministic. The same holds for all the steps involved in the two scenarios related to EGFR-induced phosphorylation, namely ERK-mediated and ligand-dependent nuclear translocation of PRB.

For the first scenario, we considered the ERK-mediated nuclear translocation of 95% of cytoplasmic PRB molecules after 5 min^4^. We assumed 66% of activated cytoplasmic ERK molecules to translocate to the nucleus within 5 min, considering an idealized cell in which activated ERK is initially cytoplasmic only, also considering that overexpressed ERK accumulates in the nucleus about 1.5–2 times its concentration in the cytoplasm^38^. For the second scenario, we considered ligand-mediated nuclear translocation of 95% of cytoplasmic PRB molecules after 30 min^8^. This defines rates k_2_, k_4_, k_6_ and k_7_ in the model, while all reactions included in this first part of the model are outlined in Table 3 and consistent with models in^39^. Molecular species in chemical reactions have the following notation: subscript $cyt$ indicates the chemical species is in the cytoplasm, while subscript $nuc$ indicates the chemical species is in the nucleus. A $p$ directly preceding a chemical species denotes it has been phosphorylated, while $b$ denotes the chemical species is bound to p4 ligand. Lastly, $ub$ stands for ubiquitin.

**Table 3:** Reactions considered in all models (‘sc’ indicates scenario).

Reaction	Region	Notes	Model
$1{:} \; {ERK}_{cyt} \stackrel{{k}_{1}}{\to } p{ERK}_{cyt}$	Cytoplasm	Lump phosphorylation, following model in^30^	1, sc 1
$2{:} \; p{ERK}_{cyt} \stackrel{k2}{\to } p{ERK}_{nuc}$	Cytoplasm	Nuclear translocation (~ 5 min)	1, sc 1
$3{:} \; {{PRB}_{cyt}+ pERK}_{cyt} \stackrel{k3}{\to } {{pPRB}_{cyt}+ ERK}_{cyt}$	Cytoplasm	PRB phosphorylation	1, sc 1
$4{:} \; {pPRB}_{cyt} \stackrel{k4}{\to } {pPRB}_{nuc}$	Cytoplasm	Nuclear translocation, (~ 5 min)	1, sc 1
$5{:} \; {pPRB}_{cyt}+p4 \stackrel{k5}{\to } {bpPRB}_{cyt}$	Cytoplasm	Ligand bound PRB	1, sc 2
$6{:} \; {bpPRB}_{cyt} \stackrel{k6}{\to } {bpPRB}_{nuc}$	Cytoplasm	Nuclear translocation, (~ 30 min)	1, sc 2
$7{:} \; {ERK}_{nuc} \stackrel{k3}{\to } {ERK}_{cyt}$	Nucleus	ERK translocation for activation (~ 20 min)	1, sc 1
$8{:} \; {PRA}_{nuc}+p{ERK}_{nuc} \stackrel{k8}{\to } {{pPRA}_{nuc}+ERK}_{nuc}$	Nucleus	PRA phosphorylation	2, sc 1
$9{:} \; p{PRB}_{nuc} + p4 \stackrel{k9}{\to } {bpPRB}_{nuc}$	Nucleus	pPRB binding to ligand	2, sc 1
$10{:} \; p{PRA}_{nuc} + p4 \stackrel{k10}{\to } {bpPRA}_{nuc}$	Nucleus	pPRA binding to ligand	2, sc 1
$11{:} \; {PRB}_{nuc} + p4 \stackrel{k12}{\to } {bPRB}_{nuc}$	Nucleus	PRB binding to ligand	2, both
$12{:} \; {PRA}_{nuc} + p4 \stackrel{k12}{\to } {bPRA}_{nuc}$	Nucleus	PRA binding to ligand	2, both
$13{:} \; {bPRA}_{nuc} + {bPRA}_{nuc} \stackrel{k13}{\to } TFAA$	Nucleus	TFAA formation	2, both
$14{:} \; {bPRA}_{nuc} + {bPRB}_{nuc} \stackrel{k14}{\to } TFAB$	Nucleus	TFAB formation	2, both
$15{:} \; {bPRB}_{nuc} + {bPRB}_{nuc} \stackrel{k15}{\to } TFBB$	Nucleus	TFBB formation	2, both
$16{:} \; {bpPRA}_{nuc} + {bpPRA}_{nuc} \stackrel{k16}{\to } TFAA$	Nucleus	TFAA formation	2, sc 1
$17{:} \; {bpPRA}_{nuc} + {bpPRB}_{nuc} \stackrel{k17}{\to } TFAB$	Nucleus	TFAB formation	2, sc 1
$18{:} \; {bpPRB}_{nuc} + {bpPRB}_{nuc} \stackrel{k18}{\to } TFBB$	Nucleus	TFBB formation	2, sc 1
$19{:} \; {bpPRA}_{nuc} + {bPRA}_{nuc} \stackrel{k19}{\to } TFAA$	Nucleus	TFAA formation	2, sc 1
$20{:} \; {bpPRA}_{nuc} + {bPRB}_{nuc} \stackrel{k20}{\to } TFAB$	Nucleus	TFAB formation	2, sc 1
$21{:} \; {bpPRB}_{nuc} + {bPRA}_{nuc} \stackrel{k20}{\to } TFAB$	Nucleus	TFAB formation	2, sc 1
$22{:} \; {bpPRB}_{nuc} + {bPRB}_{nuc} \stackrel{k21}{\to } TFBB$	Nucleus	TFBB formation	2, sc 1
$23{:} \; pPRB \left(bpPRB\right)+ub \stackrel{{k}_{23}}{\to } ub$	Nucleus	pPRB (or bpPRB) ubiquitination	2, sc 1
$24{:} \; pPRA \left(bpPRA\right)+ub \stackrel{{k}_{24}}{\to } ub$	Nucleus	pPRA (or bpPRA) ubiquitination	2, sc 1

As noted before, we used a deterministic approach for model 1, and we considered the following initial uniformly distributed numbers of molecules: *EGFR* = 50,000 as part of lumped reaction 1, *ERK*_*cyt*_ = 50,000, ${PRB}_{cyt}=3000$, and $p4$ = 36,540 (equivalent to 10^–7^ M within the cytoplasmic volume), where all other species are reaction products and, hence, were considered to have an initial null concentration. Numerical solutions at this level provided accurate estimates of translocation rates to be used in the next subsection as well as control benchmarking of the spatial simulator, on conditions of spatial homogeneity.

### Inhomogeneous distribution of PRB may accelerate ubiquitination and degradation

Reports and experiments indicate that in normal cells, the presence of high progesterone levels results in an increased intra-nuclear clustering of progesterone receptors, suggesting that PR distributions are hormonally regulated. Specifically, PRA and PRB have been observed to be evenly distributed in the proliferative phase of the menstrual cycle, and clustered into discrete foci in the secretory phase. In contrast, in endometrial cancer cells, PRA has been observed to be predominantly evenly distributed in the nucleus whereas PRB is frequently observed in intranuclear clusters^1^. Thus, the main difference between the distributions noted in the normal and cancerous cells is the lack of an even distribution of PRB in the nucleus of cancer cells in the presence of progesterone ^1^. The clusters have been described as foci, where PRB was present more than PRA and where 33% of the cases contained more than six foci per nucleus. This suggests that PRB is the principal isoform in nuclear foci. It also has been reported that when PR are in clusters, these are generally localized in regions of active chromatin, suggesting they represent transcriptionally active PR^7^. In normal cells, the median length of each foci was observed to be 0.75 μm with an interquartile range of 0.65–1.5 μm^1^, whereas in cancer tissues clusters exhibit a median length of 1.04 μm with an interquartile range of 0.78–1.94 μm^7^. It has been further suggested the difference in size corresponds to alterations in chromatin structure, which in turn allows for clusters to comprise a larger number or different complement of proteins than in normal tissues^7^.

There are other significant differences between PR clusters in normal and cancer tissues, other than their median size: their formation reliance on serum progesterone, and the ratio of homo- and hetero-dimers. A notable difference between normal tissue and cancer in PR formation is the relative distribution of PRA and PRB in foci, where both PRA and PRB form clusters in normal tissues but in endometrial cancers PRB was more common than PRA in foci^1^. Together with results in^7^, it was suggested there is aberrant PR cluster formation in cancer, compared to normal cells, which is likely impact PR-mediated transcription. Our model and simulations explore this hypothesis in detail.

For the second model, summarized by corresponding reactions in Table 3, we incorporated the translocation rates and initial conditions from the first model, and compared clustered active PR dimers in both scenarios, namely ERK-mediated and ligand-dependent nuclear translocation of PRB. Due to spatial organization, only stochastic simulations were performed for this part of the model and these used the same concentration of molecular species and reaction set rate constants considered in the deterministic model, along with the corresponding reactions outlined in Table 3 and the following additional initial numbers of molecules (uniformly distributed): ${PRB}_{cyt}={PRB}_{nuc}=3000$, ${ERK}_{cyt}=\mathrm{50,000}$, ${PRA}_{nuc}=6000$, $p4=\mathrm{15,610}$ (equivalent to 10^–7^ M within the nucleus). With the reaction and diffusion parameters in hand, a set of 20 stochastic simulations was performed with ChemCell and stochastic variation envelopes were obtained, confirming variability could be contained within the pre-defined confidence level (99%). Consistent with ChemCell definitions, all stochastic simulations were performed with particles moving within a small cube surrounding each of their current location. Such movement is considered Brownian, in the sense that the new location is sampled from a Gaussian distribution that is truncated to fit within the cube, the size of which is determined by the diffusion coefficient of the particle. The maximum probability for reaction was set to be ${P}_{max}= 0.5$ and a uniform time step of 0.001 s was used. Results were collected every 50 time steps, from which variation bounds were constructed for a total simulation time of 30 s, noting all variation envelopes were constructed with a confidence level of 99%, controlling accuracy at all time points at that level. We note that, even though individual confidence levels need not be equal (as long as their sum equals $1-\alpha$), equal confidence levels were used for all time steps since there was no time step for which variations in the simulations seemed to be considerably larger than the rest. We also note we only needed a relatively low number of simulations, as opposed to hundreds, given use of our variation bounds method.

Considering these facts, two types of simulations representing active PR dimers formation and their subsequent shuttling to clusters were performed. Since cell environments with 4–6 clusters are most common^7^, we performed simulations with 6 spherical clusters, with identical length of 1.04 μm. The active PR dimers clusters were centered at the following locations, in $\mu m$ units: (± 2.95, 0, 0), (0 ± 2.95, 0), and (0, 0 ± 2.95). Even though total TF was observed to be slightly higher in the ERK-mediated PRB nuclear translocation case, clustered TFAB was found to be roughly half of that in the ligand-induced PRB nuclear translocation case, after only 30 s. This is relevant, as heterodimeric TF seems to account for 88.5% of gene transcriptions, with PRA and PRB dimers accounting for only 3.5% and 8%, respectively.

### PR clusters influence transcription

Even though progestin treatment can reverse pre-malignant endometrial hyperplasia, the effect in endometrial cancer is far less successful. In advanced cancers, loss of PR leads to insensitivity to progestin and hormone independent growth. At the cellular level, progesterone acts through its receptors PR inducing cellular differentiation and stopping uncontrolled proliferation, inhibiting the growth of endometrial cancer^3^. Aside, PR phosphorylation via the EGFR pathway leads to ubiquitination, which could explain why endometrial cancer cells preferentially lose PR and do not respond to progestin therapy. Therefore, other than stopping uncontrolled proliferation, blocking the EGFR pathway may also result in higher levels of PR and sensitivity to progestin adjuvant therapy.

For this reason, obtaining an optimal progesterone transcription level is important and could set the stage for effective precision dual therapy with progestin and a tyrosine kinase inhibitor in endometrial cancer patients. This is an approach that, albeit not yet personalized, has started to attract attention and bear fruits^40,41^, while Systems biology methods could help find conditions to activate and protect enough PR from ubiquitination, promoting maximal transcription and allowing for progestin treatment.

Our next goal was then to simulate mRNA transcription considering clustered progesterone receptors. To do so, we explored two vastly different possibilities in cancerous cells, and compared them to the uniformly distributed case, representing normal cells. For these stochastic spatial simulations, we used the reaction set and rates described in Table 4, and ran 50 stochastic simulations for each case, to remain within the pre-defined confidence level (99%).

**Table 4:** Reactions and rates considered in the transcription model.

Reaction	Region	Notes
$1{:} \; ERK \stackrel{{k}_{1}}{\to } {ERK}_{a}$	Lumped	Lumped ERK activation
$2{:} \; PRA+p4 \stackrel{{k}_{2}}{\to } bPRA$	Nucleus	Ligand bound PRA
$3{:} \; PRB+p4 \stackrel{{k}_{3}}{\to } bPRB$	Nucleus	Ligand bound PRB
$4{:} \; bPRA+{ERK}_{a} \stackrel{{k}_{4}}{\to } {PRA}_{a}+ERK$	Nucleus	PRA activation
$5{:} \; bPRB+{ERK}_{a} \stackrel{{k}_{5}}{\to } {PRB}_{a}+ERK$	Nucleus	PRB activation
$6{:} \; {PRA}_{a} + {PRA}_{a} \stackrel{{k}_{6}}{\to } TFAA$	Nucleus	TFAA formation
$7{:} \; {PRA}_{a} + {PRB}_{a} \stackrel{{k}_{7}}{\to } TFAB$	Nucleus	TFAB formation
$8{:} \; {PRB}_{a} + {PRB}_{a} \stackrel{{k}_{8}}{\to } TFBB$	Nucleus	TFBB formation
$9{:} \; DNA + TFAA \stackrel{{k}_{9}}{\to } DNA-TFAA$	Nucleus	TFAA binding to DNA
$10{:} \; DNA + TFAB \stackrel{{k}_{10}}{\to } DNA-TFAB$	Nucleus	TFAB binding to DNA
$11{:} \; DNA + TFBB \stackrel{{k}_{11}}{\to } DNA-TFBB$	Nucleus	TFBB binding to DNA
$12{:} \; DNA-TFAA \stackrel{{k}_{12}}{\to } {DNA}_{i} + {mRNA}_{i}$	Nucleus	Lumped transcription, mRNA initiation
$13{:} \; DNA-TFAB \stackrel{{k}_{13}}{\to } {DNA}_{i} + {mRNA}_{i}$	Nucleus	Lumped transcription, mRNA initiation
$14{:} \; DNA-TFBB \stackrel{{k}_{14}}{\to } {DNA}_{i} + {mRNA}_{i}$	Nucleus	Lumped transcription, mRNA initiation
$15{:} \; {DNA}_{i} \stackrel{{k}_{15}}{\to } DNA$	Nucleus	DNA site again available for binding
$16{:} \; TFAA + ub \stackrel{{k}_{16}}{\to } ub$	Nucleus	TFAA ubiquitination
$17{:} \; TFAB + ub \stackrel{{k}_{17}}{\to } ub$	Nucleus	TFAB ubiquitination
$18{:} \; TFBB + ub \stackrel{{k}_{18}}{\to } ub$	Nucleus	TFBB ubiquitination
$19{:} \;DNA- TFAA + ub \stackrel{{k}_{19}}{\to } ub$	Nucleus	TFAA ubiquitination
$20{:} \;DNA- TFAB + ub \stackrel{{k}_{20}}{\to } ub$	Nucleus	TFAB ubiquitination
$21{:} \;DNA- TFBB + ub \stackrel{{k}_{21}}{\to } ub$	Nucleus	TFBB ubiquitination
$22{:} \; {mRNA}_{i} \stackrel{{k}_{22}}{\to } {mRNA}_{e}$	Nucleus	mRNA elongation
$23{:} \; {mRNA}_{e} \stackrel{{k}_{23}}{\to } mRNA$	Nucleus	Final mRNA

We will refer to the first case as ‘storage’, corresponding to clusters where only proportions of PRA and PRB are localized, with concentrations of 10 and 90% respectively, matching experimentally observed high concentrations of PRB, as described above. All other species were not considered localized, and the rest of the PR were evenly distributed throughout the nucleus. To evaluate the ‘storage’ hypothesis, we considered the following initial numbers of uniformly distributed particles: 2500 ERK, 2500 ERKa, 160 p4, 160 ub, and 150 hypothetical DNA binding sites. The reaction rates considered for this reaction set were k_1_ = k_15_ = 1, k_12_ = 0.0355, k_13_ = 0.8852, k_14_ = 0.0793, k_22_ = 30, k_23_ = 1, and k_j_ = 10^10^ for all binary reactions. These reactions and rates followed experimental findings referenced in Table 1 and^38,42–48^. Rates k_12_, k_13_ and k_14_ are order 1, multiplied times the fraction that corresponds to the transcription of homodimers vs heterodimers (see “Discussion”). We note, however, that some tested variations in k_12_, k_13_ and k_14_ did not significantly change conclusions derived from our simulations, when these three rates summed to 1. We further simulated cell environments with 4, 7 and 15 clusters; namely, at or above typical cell environments with 4–6 clusters, as experimentally observed and explained above. For the case with 4 foci, each cluster contained 63 molecules of PRA and 563 of PRB, while the remaining 2248 molecules of PRA and 248 molecules of PRB were uniformly distributed throughout the nucleus. The simulations representing 15 clusters contained 16 molecules of PRA and 150 of PRB inside each well-spaced compartment, while the remaining 2260 molecules of PRA and 250 of PRB were uniformly distributed throughout the nucleus. We performed simulation sets with various clusters radii but, in all cases, mRNA production considering 4–6 clusters was found to be lower than in the uniformly distributed case, and significantly lower with 15 foci.

To assess a scenario in which focal distribution could enhance mRNA transcription, we considered a second possibility with all reaction products from PR down to TF to be clustered. We call this the ‘spots’ scenario where, once TF molecules are formed, they can diffuse and bind to nearby DNA sites promoting transcription. For these simulations one should keep in mind that all molecules are close to one another, tightly packed, and therefore reactions can happen rather quickly. One interesting question for this scenario would be if and how transcription varies according to the distance between DNA binding sites and the cluster, keeping in mind the dependency on diffusion rates. For such a purpose we derived a simplified model with a single cluster, considering cluster behavior is independent of the others. For these simulations we adopted 1000 PRB, 1000 p4, and one DNA binding site as initial numbers of molecules, and we studied varying distances from the cluster, assuming two different diffusion coefficients (10^–8^ and 10^–9^ cm^2^/s). Strikingly, our numerical simulations returned as optimal value the exact same cluster radius as experimentally observed in^1^. We also observed that, for a diffusion coefficient of 10^–9^ cm^2^/s, typical for a macromolecule inside the nucleus, the optimal distance of a DNA binding site to the cluster center is 0.75 μm. Considering these results, we then constructed a final simulation set with 4 clusters and DNA binding sites localized in their boundaries, from which we observed that if all progesterone active forms are kept clustered, transcription is even lower than the ‘storage’ and the uniformly distributed case.

## Discussion

In this work, we hypothesized that optimal progesterone transcriptional activity may require nearly equal molarities of PRA and PRB inside the nucleus, optimizing the formation of the PRA-PRB heterodimer instead of homodimers of PRA or PRB. In the context of endometrial cancer, this is a potential ideal scenario that would titrate growth factor activation of MAP Kinase activity to promote phosphorylation of both PR isoforms, inducing translocation of phosphorylated PRB to the nucleus and formation of ligand-bound heterodimers, without loss of most active dimers to ubiquitin-mediated degradation. To test this, we built data-based quantitative spatiotemporal mechanistic models, numerically solved deterministic parts, and simulated related stochastic reactions. It is worth noting one alternative to spatially resolved models is deriving time delays for translocation processes and using the delay chemical master equation to simulate the entire system (see for instance^13^). However, in doing so one cannot retain spatial resolution, which was needed in this study. We also presented a method we developed to assess variations in stochastic simulations over the entire simulation timespan, which also helps define the number of necessary stochastic simulations to guarantee accuracy at a pre-defined level (in our case 99%).

Overall, our simulations showed that focal distributions of receptors in conjunction with blocked EGFR activity may result in a higher production of heterodimeric transcription factors, which in turn seems responsible for approximately 88.5% of the total gene regulation in single cells. The numerical results support the hypothesis that cancerous cells have lower transcription levels based on overexpression of EGFR. These results assume PR to be vulnerable targets of ubiquitination as a result of phosphorylation on serine 294, while being “protected” from ubiquitination once the active PR dimers are formed. We do note that this may not be necessarily the case and, ultimately, assuming the latter can only result in any other possible scenario having lower transcriptional activity. It is left for biological experimentation to validate and confirm whether the observed active PR dimers being shuttled to a cluster are still vulnerable for ubiquitination or not.

Importantly, our simulations also showed that focal distributions of receptors result in lower transcription. This may seem counterintuitive at first as one would expect a higher transcription rate to be a direct consequence of PR proximity. PR however, cannot be activated unless binding to progesterone ligands and ERK, both of which are well mixed, resulting in a lower concentration of active receptors in time. In other words, faster collisions in clusters do not necessarily imply higher or faster transcription, e.g. when clusters are away from DNA binding sites and active transcription factors must travel longer than in uniformly distributed cases. On the other hand, and depending on rates, receptor proximity could allow for faster production of active transcription factors. In this case, ubiquitin could not destroy the active forms of progesterone receptor as easily, thereby promoting higher transcription, due to their distribution. We speculate the first two processes to be the limiting steps for transcriptional products, since fewer active forms of progesterone are produced and all subsequent steps depend on these products. The model was also extended to account for differences in the initial distribution of PR, representing typical scenarios of normal and endometrial cancerous cells.

As a side note, unique sets of genes up or down-regulated by PRA-PRA, PRB-PRB, and PRA-PRB have been mapped in Hec50co endometrial cancer cells and, from these, endometrial cancer cells expressing both PR isoforms (where heterodimers are possible) have reportedly shown about a tenfold more robust genomic response to progesterone, with over 10× genes found to be regulated by PRA + PRB when compared to the number of genes regulated by PRB and PRA alone (communications with Dr. K. Leslie, also see^12^ and references therein). Additional transcriptional differences have been reported in the literature^2^ and we also refer interested readers to^12,31,49^ and citations therein. Hence, optimal progesterone transcriptional activity may require nearly equal molarities of PRA and PRB inside the nucleus.

Altogether, our numerical results support the hypothesis that clustered receptors, as found in cancerous cells, result in lower transcription rates. This is of particular interest since ubiquitination was thought to be the only process that limited transcription. Therefore, our simulations based on experimental measurements support a second hypothesis that may be closer to the biology. Namely, that receptor clustering itself can hinder transcription.

There are some model limitations worth pointing out. First, our results are based on single-cell dynamics alone. Also, our model assumes dimerization based on various experimental observations and models published in the literature, such as^50–55^, but dimerization is not always essential for transcription, as noted in^56^. For instance, in human endometrial stromal cells, overexpression of PRA or PRB can mediate the transcription of thousands of genes when the endogenous PR is knocked down^57^. In mice, PR monomers have been observed to interact with DNA that has the half-site of progesterone response element^58^ while, also, in mice, it has been reported that the deletion of PRA but not PRB disrupts uterine functions^59,60^. Results from various model organisms and context could indicate heterodimers need not influence the most genes in general, but rather that PRA, PRB, homodimers and heterodimers mediate transcription in a context and model-organism dependent manner. Therefore, results from our systems biology model and simulations in individual Hec50co cells are subject to the assumptions and experimental data referenced in this study, while generalization from single cells to broad actions requires targeted experiments in multi-cellular contexts, tissue samples, and biochemical contexts. Lastly, it would also be interesting to quantify and validate levels of blocked EGFR through a tyrosine kinase inhibitor, which could potentially reveal if any such blockage level can achieve higher transcription than ligand-induced nuclear translocation of PRB alone, a matter that falls outside the scope of our presented model but that would be necessary for precision therapeutics.

## Data Availability

All data used in this work are publicly available.

## Abbreviations

PR: Progesterone receptor
PRA: Progesterone receptor subtype A
PRB: Progesterone receptor subtype B
p4: Progesterone ligand
ERK: Extracellular-signal-regulated kinase
EGFR: Epidermal growth factor receptor
ub: Ubiquitin
TF: Transcription factor (ligand-bound PR dimer)
TFAA: TF from PRA homodimer
TFBB: TF from PRB homodimer
TFAB: TF from PRA-PRB heterodimer
CLT: Central limit theorem
